# Parthenolide Phytosomes Attenuated Gentamicin-Induced Nephrotoxicity in Rats via Activation of Sirt-1, Nrf2, OH-1, and NQO1 Axis

**DOI:** 10.3390/molecules28062741

**Published:** 2023-03-17

**Authors:** Rawan S. Albalawi, Lenah S. Binmahfouz, Rawan H. Hareeri, Rasheed A. Shaik, Amina M. Bagher

**Affiliations:** Department of Pharmacology and Toxicology, Faculty of Pharmacy, King Abdulaziz University, Jeddah 21589, Saudi Arabia

**Keywords:** gentamicin, nephrotoxicity, parthenolide, phytosomes, KIM-1, NFκB, Sirt-1, Nrf2

## Abstract

Nephrotoxicity is a serious complication that limits the clinical use of gentamicin (GEN). Parthenolide (PTL) is a sesquiterpene lactone derived from feverfew with various therapeutic benefits. However, PTL possesses low oral bioavailability. This study aimed to evaluate the therapeutic protective effects of PTL-phytosomes against GEN-induced nephrotoxicity in rats. The PTL was prepared as phytosomes to improve the pharmacological properties with a particle size of 407.4 nm, and surface morphology showed oval particles with multiple edges. Rats were divided into six groups: control, nano-formulation plain vehicle, PTL-phytosomes (10 mg/kg), GEN (100 mg/kg), GEN + PTL-phytosomes (5 mg/kg), and GEN + PTL-phytosomes (10 mg/kg). The administration of PTL-phytosomes alleviated GEN-induced impairment in kidney functions and histopathological damage, and decreased kidney injury molecule-1 (KIM-1). The anti-oxidative effect of PTL-phytosomes was demonstrated by the reduced malondialdehyde (MDA) concentration and increased superoxide dismutase (SOD) and catalase (CAT) activities. Furthermore, PTL-phytosomes treatment significantly enhanced sirtuin 1 (Sirt-1), nuclear factor erythroid-2-related factor-2 (Nrf2), NAD(P)H quinone dehydrogenase 1 (NQO1), and heme oxygenase-1 (HO-1). Additionally, PTL-phytosomes treatment exhibited anti-inflammatory and anti-apoptotic properties in the kidney tissue. These findings suggest that PTL-phytosomes attenuate renal dysfunction and structural damage by reducing oxidative stress, inflammation, and apoptosis in the kidney.

## 1. Introduction

Drug nephrotoxicity is a health problem worldwide, with high mortality and morbidity in adults and pediatric patients. Approximately 20% of acute kidney injury in hospitalized patients is attributed to drug toxicity [1,2]. Furthermore, the detection of nephrotoxicity is often delayed until a noticeable reduction in renal function has occurred [3]. Gentamicin (GEN) is an aminoglycoside antibiotic that is popularly used because of its ability to treat infections with gram-negative aerobes, its synergistic effect with other antibiotics, and its low cost [4,5]. Nonetheless, nephrotoxicity associated with GEN treatment is a devastating complication in the clinical setting [6]. Up to 30% of patients receiving GEN therapy for more than seven days experience kidney impairment [7]. Moreover, even a single dose and the lowest therapeutic doses of GEN can cause acute kidney injury [8,9].

GEN-induced nephrotoxicity represents a complicated phenomenon manifested by elevated serum creatinine and urea levels [8]. This is associated with the accumulation of GEN in the proximal renal tubules, causing tubular necrosis [10]. The exact mechanism of GEN-induced nephrotoxicity is not well understood [11]. One major contributing factor to the pathophysiology of GEN-induced nephrotoxicity is oxidative stress [12]. Evidence suggests that GEN therapy causes oxidative stress by increasing reactive oxygen species (ROS) production, by reducing the efficacy of antioxidant enzymes, and by peroxidation of the lipid membrane. Furthermore, it has been demonstrated that the accumulation of oxidative stress causes inflammation, apoptosis, and kidney injury [13].

Overall, compounds with antioxidant, anti-inflammatory, and anti-apoptotic properties play a significant protective role in nephrotoxicity by GEN [14]. Recently, there has been much interest in exploring the effects of naturally occurring dietary ingredients in preventing GEN-induced nephrotoxicity [12,13,15]. Sesquiterpene lactones are active ingredients found in traditional medicinal plants that act as antimicrobial, antimigraine, and anti-inflammatory agents, as well as for treating stomachaches and skin infections [16]. Parthenolide (PTL) is a sesquiterpene lactone present in the feverfew plant (Tanacetum parthenium) that has long been used in traditional medicine [17]. Feverfew and PTL have been integrated into various functional health supplements [18,19]. Interestingly, it has been demonstrated that PTL exhibits multiple activities, including antioxidative and anti-inflammatory effects [20]. Previous research has shown that PTL alleviates cisplatin-induced renal damage in rats [21].

The anti-oxidative effects of PTL can be attributed to the inhibition of malondialdehyde (MDA) concentration [21] and ROS production, as well as increasing antioxidant enzyme activity, including superoxide dismutase (SOD) [20]. In addition, it has been proven that the regulation of ROS production by PTL is linked to the regulation of the nuclear factor erythroid 2-related factor (Nrf2)-Kelch-like ECH-associated protein 1/(Keap1) pathway [18]. The Nrf2-Keap1 pathway is the primary regulator of cytoprotective responses to oxidative and electrophilic stress [22]. Nrf2 plays a significant role in regulating the expression of several cytoprotective genes, such as those encoding antioxidant and detoxifying enzymes (phase II) such as catalase (CAT), SOD, NAD(P)H quinone dehydrogenase 1 (NQO1), and heme oxygenase-1 (HO-1). Thus, Nrf2 and its target genes represent an essential defense against oxidative stress [23]. Crosstalk between silent information regulator sirtuin 1 (Sirt-1) and the Nrf2-Keap1 anti-oxidative pathway has been previously reported [24]. Sirt1 is a regulator upstream of the Nrf2 signaling pathway; it is upregulated in response to oxidative stress [25]. A substantial body of research demonstrated that Sirt1 and Nrf2/HO-1expression was downregulated after GEN treatment. Targeting this pathway has been proposed to be a possible protective route against GEN-induced nephrotoxicity [25,26,27].

PTL has been demonstrated to suppress the activation of pro-inflammatory cytokines interleukin-6 (IL-6), tumor necrosis factor-alpha (TNF-α), and the nuclear translocation of nuclear transcription factor-kappa B (NF-κB) [28]. NF-κB signaling contributes to the initiation and development of inflammatory diseases by activating pro-inflammatory cytokines. Indeed, the activation of NF-κB signaling in renal cells is linked to experimental and human kidney diseases. Consequently, downregulation of the NF-κB pathway plays a central role in preventing inflammation response [29,30]. Eventually, PTL could inhibit apoptosis mediated by oxidative stress and inflammation [31,32].

However, PTL has low solubility and bioavailability, limiting its in vivo application [33,34]. Previous studies have shown that the bioavailability and solubility of PTL can be significantly increased by many drug delivery systems [35,36,37,38]. Phytosomes serve as a nano-delivery system of phytoconstituents to enhance oral absorption, lipid solubility, and biological membrane crossing, resulting in improved bioavailability [39,40]. Hence, this study aimed to investigate the protective effects of PTL-phytosomes in GEN-induced nephrotoxicity in a rat model.

## 2. Results

### 2.1. Preparation and Characterization of PTL-Phytosomes

**Figure 1:**
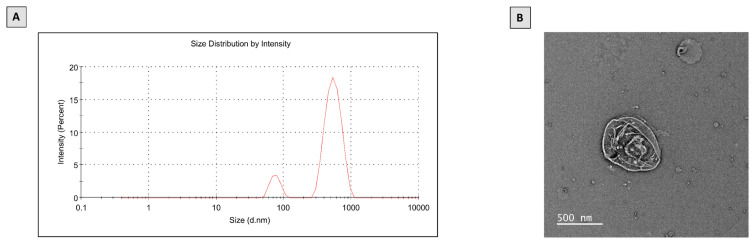
Characterization of PTL-phytosomes. (**A**) Particle size distribution, (**B**) TEM image.

Initially, we investigated the features of the prepared PTL-phytosomes, including size and structural morphology (Figure 1). The prepared phytosomes show a z-average size of 407.4 and a polydispersity index (PDI) of 0.600 (Figure 1A). PDI, ranging from 0 to 1, is considered an indicator of the homogeneity of size distribution. The smaller the PDI, the more homogenous the nanoparticulate formulation. PDI values of less than 0.05 indicate monodispersed samples, PDI of less than 0.3 are considered to have narrow and homogenous size distribution, while values >0.7 indicate a broad-sized system [41,42,43]. Accordingly, the prepared phytosomes could have acceptable homogeneity. Furthermore, the PTL-phytosomes were characterized by Transmission Electron Microscopy (TEM) analysis (Figure 1B). The TEM photograph of the prepared PTL-phytosomes revealed that the phytosome’s surface morphology showed oval particles with multiple edges.

### 2.2. PTL-Phytosomes Attenuated GEN-Induced Kidney Hypertrophy in Rats

**Figure 2:**
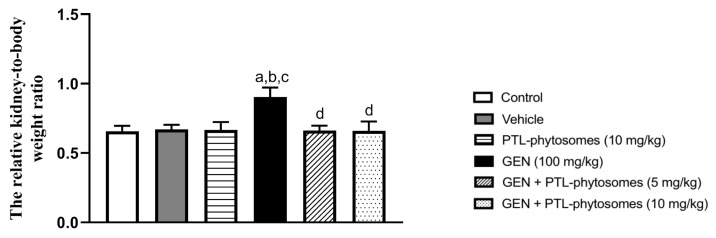
PTL-phytosomes treatment improved the relative kidney-to-body weight ratio. Data are expressed as Mean ± SD (*n* = 6). a: significant when compared to control at *p* < 0.05; b: significant when compared to vehicle at *p* < 0.05; c: significant when compared to PTL-phytosomes at *p* < 0.05; d: significant when compared to (GEN)-alone at *p* < 0.05.

To determine the beneficial effects of PTL-phytosomes treatment in attenuating GEN-induced kidney hypertrophy, we calculated the relative kidney-to-body weight ratio for all treatment groups. As demonstrated in Figure 2, there was no significant difference between the control, vehicle, and PTL-phytosomes (10 mg/kg)-treated rats. However, GEN treatment for seven continuous days increased the relative kidney-to-body weight ratio (37.4%) compared to the control, which indicated kidney hypertrophy. Interestingly, PTL-phytosomes (5 and 10 mg/kg) treatment for seven continuous days attenuated the GEN-induced increase in the kidney-to-body weight ratio (by 26.7% and 26.8%, respectively) compared to the GEN-treated group. These findings suggest that PTL-phytosomes treatment can ameliorate GEN-induced kidney hypertrophy as indicated by restoring the relative kidney-to-body weight ratio to normal levels.

### 2.3. PTL-Phytosomes Restored the Kidney Function Markers

**Figure 3:**
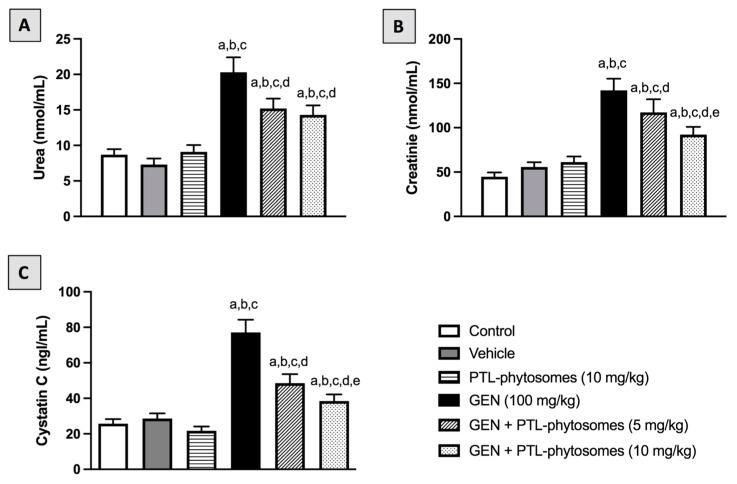
PTL-phytosomes ameliorated kidney functions in GEN-treated rats. Serum levels of (**A**): urea, (**B**): creatinine, (**C**): cystatin C were measured. Data are expressed as Mean ± SD (*n* = 6). a: significant when compared to control at *p* < 0.05; b: significant when compared to vehicle at *p* < 0.05; c: significant when compared to PTL-phytosomes at *p* < 0.05; d: significant when compared to GEN-alone at *p* < 0.05; e: significant when compared to GEN + PTL-phytosomes (5 mg/kg) at *p* < 0.05.

Next, we explored the effects of PTL-phytosomes on the serum levels of urea, creatinine, and cystatin C in GEN-treated rats (Figure 3). We observed that the treatment of vehicle and PTL-phytosomes (10 mg/kg) did not alter urea, creatinine, and cystatin C levels compared to the control. However, GEN-treated rats showed elevated serum levels of urea (133.3%), creatinine (217.9%), and cystatin C (200%) relative to the control, which indicates an impairment in kidney functions. The co-administration of PTL-phytosomes (5 or 10 mg/kg) with GEN significantly decreased the serum levels of urea (25.1% or 29.5%), creatinine (17.5% or 35.1%), and cystatin C (37.1% or 50.1%), compared to GEN-treated group.

### 2.4. PTL-Phytosomes Ameliorated, Renal Histopathological Changes, Induced by GEN Treatment

To examine the impact of PTL-phytosomes on GEN-induced histopathological alterations in kidney tissue, hematoxylin and eosin (H&E), periodic acid-Schiff (PAS), Masson’s Trichrome (MT), and Sirius Red (SR) stains were performed.

**Figure 4:**
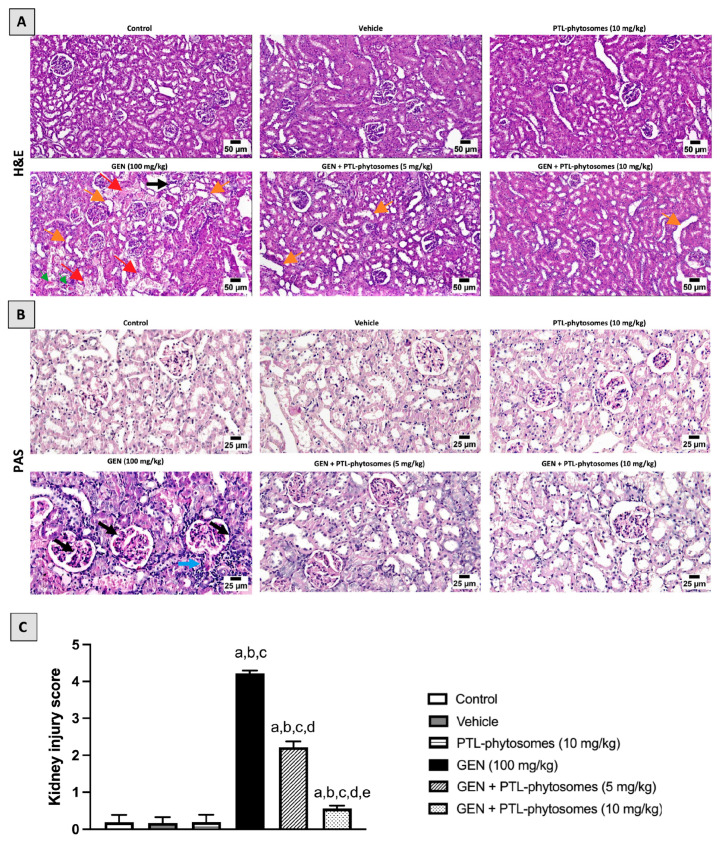
PTL-phytosomes inhibited GEN-induced kidney damage. Photomicrographs of kidney sections stained with H&E and PAS stains from different groups of experimental animals. (**A**) In the H&E-stained section, GEN-induced tubular necrosis (red arrows), peritubular inflammatory cell infiltration (black arrow), tubular dilatations (orange arrows), and cast formation (green arrowhead) were observed. Conversely, the co-treatment of PTL-phytosomes (5 or 10 mg/kg) with GEN notably improved histoarchitecture of glomerulus and tubules, except for a few tubular dilatations in kidney tissue observed (orange arrows), scale bar 50 μm. (**B**) In the PAS-stained section, GEN caused extensive structural glomerular damage (black arrows) and Bowman’s capsule rupture with inflammatory cell infiltration (blue arrow). However, these alterations were less severe in co-treatment of PTL-phytosomes (5 or 10 mg/kg) + GEN, scale bar 25 μm. (**C**) Kidney injury score was evaluated. Data are expressed as Mean ± SD (*n* = 6). a: significant when compared to control at *p* < 0.05; b: significant when compared to vehicle at *p* < 0.05; c: significant when compared to PTL-phytosomes at *p* < 0.05; d: significant when compared to GEN alone at *p* < 0.05; e: significant when compared to GEN + PTL-phytosomes (5 mg/kg) at *p* < 0.05.

In H&E and PAS stains, a photomicrograph of kidney tissue revealed the normal histological structure of renal corpuscles and tubules among three groups: the control, vehicle, and PTL-phytosomes (10 mg/kg) (Figure 4). In contrast, the GEN-alone treated group exhibited severe kidney damage, including glomerular degeneration and tubules that were significantly necrotic and cystically dilated. This was associated with moderate peritubular inflammatory cell infiltration and cast formation (Figure 4A). Tissue sections from this group revealed severe thickening of the glomerulus basement membrane and capillary tufts, resulting in glomerular hypertrophy and an increase in the mesangial matrix. In addition, Bowman’s capsule rupture was found with periglomerular-interstitium-infiltrated inflammatory cells (Figure 4B). Correspondingly, the kidney injury score was evaluated. In the GEN-treated group, the scores reached 4.2, which is much higher than the control groups (Figure 4C). However, these histopathological alterations were minimal in PTL-phytosomes (5 mg/kg) + GEN co-treatment, as evidenced by the absence of cast formation, the presence of a few cystic dilatations, and necrobiotic changes in renal tubules with fewer numbers of inflammatory cells in the interstitial tissue. The kidney injury score was decreased to 2,2, compared to GEN-treated rats (Figure 4C). Interestingly, the administration of PTL-phytosomes (10 mg/kg) with GEN showed a normal histoarchitecture of renal corpuscles and tubules. The kidney score from this group decreased to 0.5 compared to GEN-treated rats (Figure 4C).

**Figure 5:**
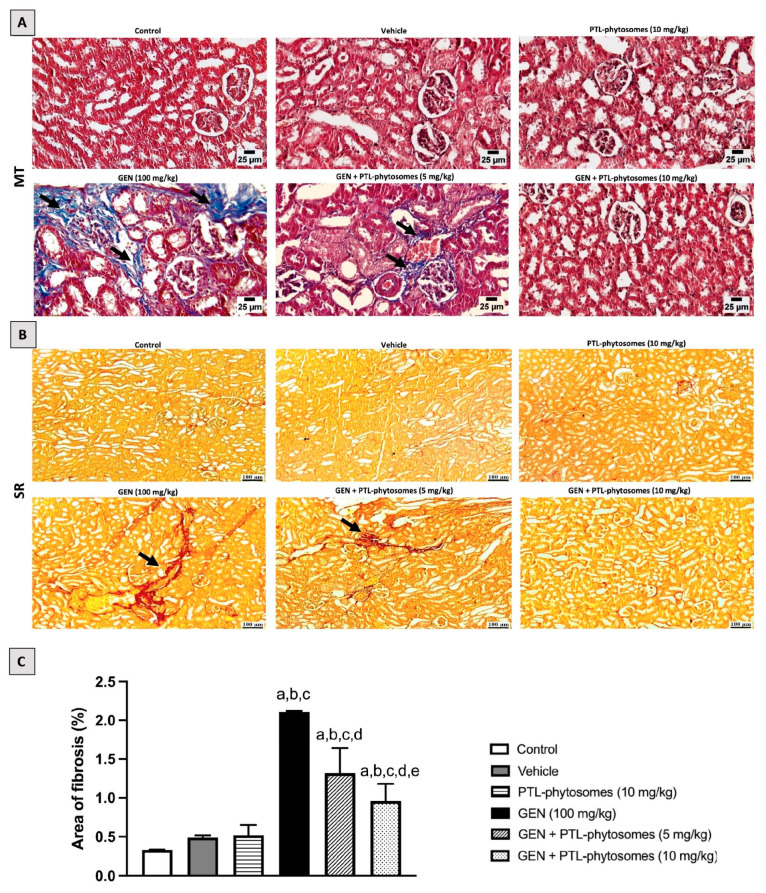
PTL-phytosomes alleviated GEN-induced kidney fibrosis. Photomicrographs of kidney sections stained with MT and SR stains to double confirm interstitial fibrosis. (**A**,**B**) In the MT- and SR-stained sections, GEN caused collagen deposition in the renal parenchyma (black arrows), characteristic of interstitial fibrosis. In contrast, treating with PTL-phytosomes (5 mg/kg) with GEN showed minimal collagen deposition. Moreover, PTL-phytosomes (10 mg/kg) + GEN co-administration showed a normal histological picture of the renal parenchyma. Scale bars, 25 and 100 μm. (**C**) Semi-quantitative assessment of renal fibrosis area. Data are expressed as Mean ± SD (*n* = 6). a: significant when compared to control at *p* < 0.05; b: significant when compared to vehicle at *p* < 0.05; c: significant when compared to PTL-phytosomes at *p* < 0.05; d: significant when compared to (GEN)-alone at *p* < 0.05; e: significant when compared to GEN + PTL-phytosomes (5 mg/kg) at *p* < 0.05.

Next, we evaluated interstitial fibrosis in kidney tissue (Figure 5A,B). MT and SR stains revealed a significant increase in collagen fibril deposition area in the GEN-treated group (2.11%), which is significantly more than in the control groups. Conversely, PTL-phytosomes (5 mg/kg) + GEN co-treatment decreased collagen fibril deposition, and the fibrosis area represents 1.32%, compared to GEN-treated rats. Interestingly, PTL-phytosomes at a 10 mg/kg dose with GEN co-treatment lowers collagen fibril deposition, and the fibrosis area represents 0.96%, relative to GEN-treated rats. These results demonstrate that the PTL-phytosomes treatment alleviated inflammation, tubular necrosis, and interstitial fibrosis in GEN-treated rats.

### 2.5. PTL-Phytosomes Ameliorated GEN-Induced Kidney Injury

**Figure 6:**
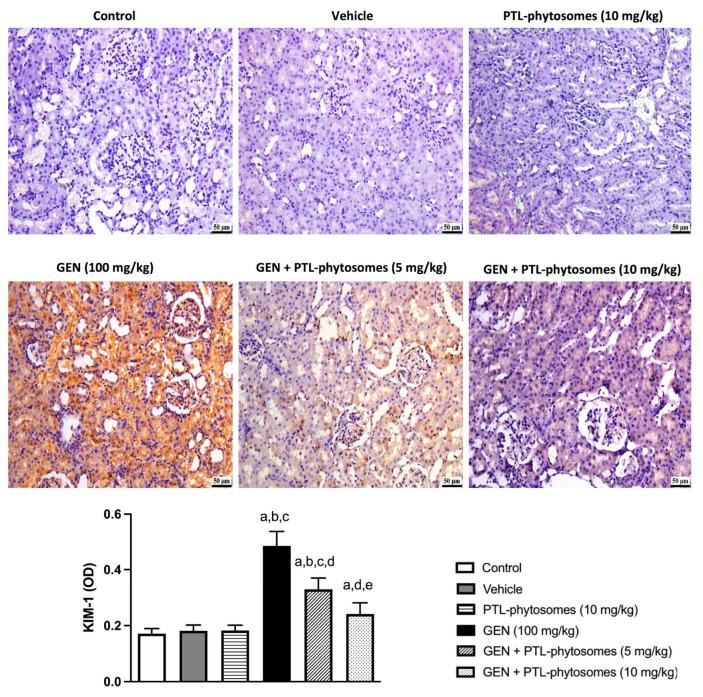
PTL-phytosomes prevented GEN-induced kidney injury by suppressing KIM-1 expression. The immunohistochemical staining section was quantified as optical density (OD). Data are expressed as Mean ± SD (*n* = 6). a: significant when compared to control at *p* < 0.05; b: significant when compared to vehicle at *p* < 0.05; c: significant when compared to PTL-phytosomes at *p* < 0.05; d: significant when compared to GEN alone at *p* < 0.05; e: significant when compared to GEN + PTL-phytosomes (5 mg/kg) at *p* < 0.05, scale bar 50 μm.

Next, to elucidate the effect of PTL-phytosomes on GEN-induced kidney injury, the expression of kidney injury molecule-1 (KIM-1) in the rat kidney was investigated. KIM-1 is a sensitive biomarker of kidney injury. As depicted in Figure 6, vehicle and PTL-phytosomes (10 mg/kg) treatment showed no significant changes in KIM-1 expression relative to the control. However, GEN treatment significantly elevated KIM-1 expression (182.5%) compared to the control group. Interestingly, the co-administration of PTL-phytosomes 5 mg/kg or 10 mg/kg significantly suppressed GEN-induced KIM-1 expression by 32.1% or 50.2 %, respectively, relative to the GEN-treated group. Thus, PTL-phytosomes treatment might be a potential candidate against GEN-induced kidney injury.

### 2.6. PTL-Phytosomes Attenuated Oxidative Stress in GEN-Treated Rats

**Figure 7:**
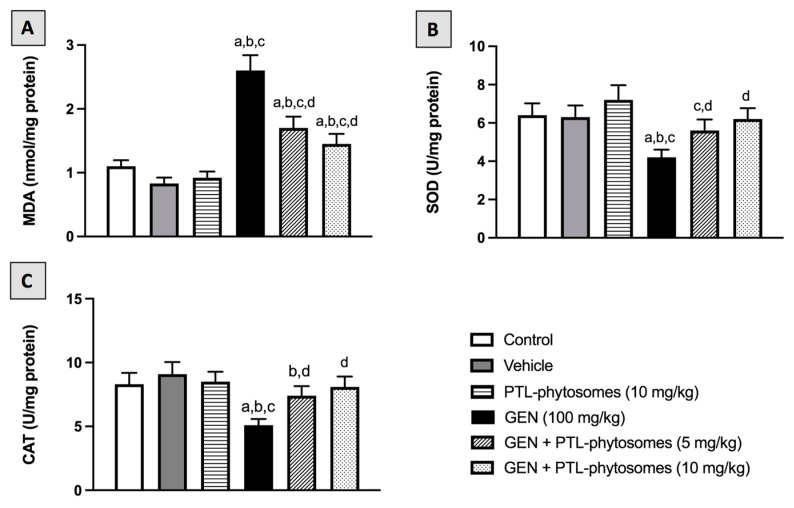
PTL-phytosomes restored oxidative stress markers in GEN-treated rats. (**A**) MDA, (**B**) SOD, and (**C**) CAT were used as oxidative status markers. Data are expressed as Mean ± SD (*n* = 6). a: significant when compared to control at *p* < 0.05; b: significant when compared to vehicle at *p* < 0.05; c: significant when compared to PTL-phytosomes at *p* < 0.05; d: significant when compared to GEN-alone at *p* < 0.05.

Next, we investigated the potential protective effects of PTL-phytosomes against GEN-induced oxidative stress in the rats. MDA, a marker of oxidative stress, was evaluated in the kidney tissues. As shown in Figure 7A, GEN considerably increased the MDA content of the kidney (136.3%) compared to the control group. Conversely, the co-administration of PTL-phytosomes at both 5 and 10 mg/kg doses significantly reduced GNE-induced increase in the kidney MDA content by (34.6%) and (46.2%), respectively, when compared to GEN-treated rats. Furthermore, the activities of the antioxidant enzymes, including SOD and CAT, were evaluated in the kidney tissues. GEN treatment substantially decreased SOD and CAT by 34.4% and 38.5% relative to the control group (Figure 7B,C). In contrast, administration of PTL-phytosomes 5 mg/kg with GEN markedly induced the activities of SOD and CAT by 33.3% and 45.1%, respectively, as compared with GEN treatment. Similarly, PTL-phytosomes at a 10 mg/kg dose increased SOD and CAT activities by 47.6% and 58.8%, respectively. Our data demonstrate that PTL-phytosomes ameliorate GEN-induced oxidative stress in rat kidneys.

### 2.7. PTL-Phytosomes Suppressed GEN-Induced Oxidative Stress via Activation of Sirt-1, Nrf2, NQO1, and OH-1 Axis

**Figure 8:**
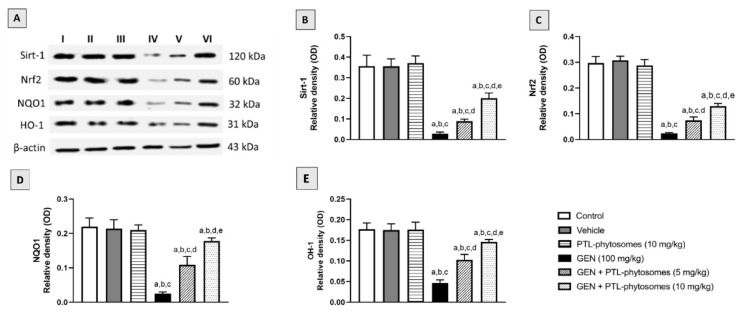
PTL-phytosomes activated the antioxidant defense system. (**A**) Western blot analysis to different experimental groups, I control, II vehicle, III PTL-phytosomes (10 mg/kg), IV GEN (100 mg/kg), V GEN + PTL-phytosomes (5 mg/kg), and (VI) GEN + PTL-phytosomes (10 mg/kg). The semi-quantification of (**B**) Sirt-1, (**C**) Nrf2, (**D**) NQO1, and (**E**) HO-1 expression was achieved. Data are expressed as Mean ± SD (*n* = 6). a: significant when compared to control at *p* < 0.05; b: significant when compared to vehicle at *p* < 0.05; c: significant when compared to PTL-phytosomes at *p* < 0.05; d: significant when compared to GEN-alone at *p* < 0.05; e: significant when compared to GEN + PTL-phytosomes (5 mg/kg) at *p* < 0.05.

To confirm the protective role of PTL-phytosomes against GEN-induced oxidative stress in rat kidneys, the protein expression of the antioxidant defense system, including Sirt1, Nrf2, (NQO1), and HO-1, was investigated (Figure 8). Significantly, GEN treatment reduced Sirt-1, Nrf2, NQO1, and HO-1 expressions by 91.8%, 91.9%, 88.6%, and 73.4%, respectively, compared to the control group. It is important that PTL-phytosomes co-treatment at 5 mg/kg with GEN increased their expression by 207.9%, 212.5%, 336%, and 119.1%, respectively, relative to GEN-treated rats. The highest protection was observed in PTL-phytosomes at 10 mg/kg + GEN, which increased their expressions by 595.5%, 441.7%, 612%, and 210.6%, respectively, compared to GEN-treated rats. These findings suggest that the PTL-phytosomes treatment could promote the antioxidant defense system in kidney rats against GEN-induced oxidative stress.

### 2.8. PTL-Phytosomes Protected the Kidney against GEN-Induced Inflammation

**Figure 9:**
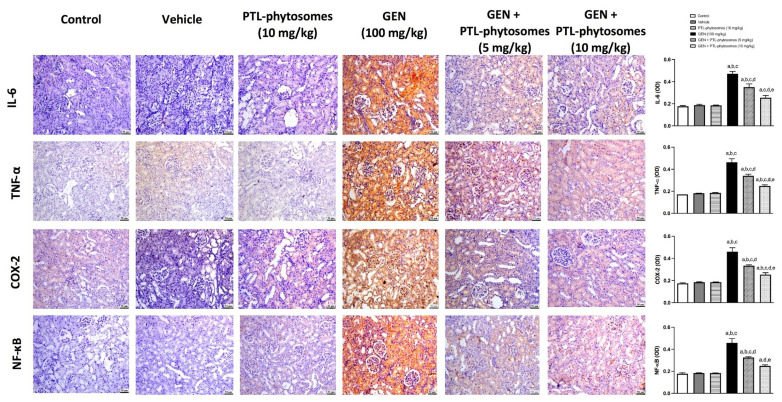
PTL-phytosomes reduced the expression of IL-6, TNF-α, COX-2, and NF-κB (p65) in GEN-treated rats. The immunohistochemical staining section was quantified as OD. Data are expressed as Mean ± SD (*n* = 6). a: significant when compared to control at *p* < 0.05; b: significant when compared to vehicle at *p* < 0.05; c: significant when compared to PTL-phytosomes at *p* < 0.05; d: significant when compared to GEN alone at *p* < 0.05; e: significant when compared to GEN + PTL-phytosomes (5 mg/kg) at *p* < 0.05. Scale bar, 50 μm.

Furthermore, we assessed the influence of PTL-phytosomes on inflammation induced by GEN treatment. Immunohistochemical staining was used to determine the levels of IL-6, TNF-α, cyclooxygenase-2 (COX-2), and NF-κB (p65) protein expression in the rat kidneys (Figure 9). Treatment of rats with PTL-phytosomes 10 mg/kg did not show any changes in the expression of these inflammatory markers. However, GEN treatment showed significantly higher IL-6, TNF-α, COX-2, and NF-κB (p65) levels by 166.1%, 169.5%, 163.4%, and 157.3%, respectively, relative to the control group. Interestingly, co-administration of PTL-phytosomes 5 mg/kg suppressed GEN-induced expression of IL-6 (25.6%), TNF-α (26.5%), COX-2 (27.3%), and NF-κB (p65) (28.9%) levels relative to GEN treated group. Similarly, co-administration of PTL-phytosomes 10 mg/kg with GEN significantly suppressed the expression of IL-6 (45.8%), TNF-α (46.3%), COX-2 (44.9%), and NF-κB (p65) (45.6%) in comparison to GEN-treated group. Overall, these results indicate that PTL-phytosomes treatment attenuated the inflammatory response induced by GEN treatment.

### 2.9. PTL-Phytosomes Ameliorated GEN-Induced Kidney Apoptosis

**Figure 10:**
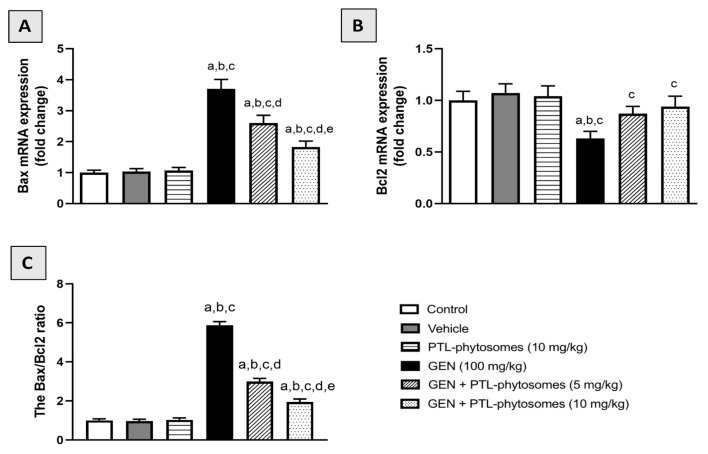
PTL-phytosomes regulated the expression of (**A**) Bax and (**B**) Bcl-2 (**C**) The Bax/Bcl-2 ratio in GEN-induced apoptosis. Data are expressed as Mean ± SD (*n* = 6). a: significant when compared to control at *p* < 0.05; b: significant when compared to vehicle at *p* < 0.05; c: significant when compared to PTL-phytosomes at *p* < 0.05; d: significant when compared to GEN-alone at *p* < 0.05; e: significant when compared to GEN + PTL-phytosomes (5 mg/kg) at *p* < 0.05.

Next, to assess the effect of PTL-phytosomes on apoptosis, the mRNA expression levels of Bcl-2-associated X-protein (Bax) and B-cell lymphoma 2 (Bcl-2) were measured using real-time polymerase chain reaction (RT-PCR). Figure 10A shows no significant differences in Bax mRNA expression were observed in the vehicle and PTL-phytosomes (10 mg/kg) groups compared with the control group. GEN treatment induced apoptosis, as shown by the significantly elevated proapoptotic Bax expression (270%) relative to the control group. However, PTL-phytosomes treatment at 5 and 10 mg/kg doses combined with GEN attenuated these changes by downregulating Bax mRNA expression by 29.7% and 50.5%, respectively, compared to GEN treatment alone. Furthermore, we investigated the mRNA expression of the anti-apoptotic Bcl-2 in kidney tissue (Figure 10B). Comparatively to the control group, GEN significantly downregulated Bcl-2 mRNA expression by 37%. The expression of Bcl-2 was upregulated considerably when PTL-phytosomes at 5 and 10 mg/kg was administered with GEN by 38.1% and 49.2%, respectively, relative to the GEN-treatment group. Moreover, the Bax/Bcl-2 ratio was markedly increased by 487.3% in the GEN-treated group relative to the control group (Figure 10C). The co-administration of PTL-phytosomes 5 and 10 mg/kg with GEN significantly decreased this ratio by 49.1% and 66.8%. Taken together, our data demonstrate that PTL-phytosomes could regulate the expression of apoptosis markers in GEN-treated rats.

## 3. Discussion

Nephrotoxicity is a significant limitation of GEN therapy [9]. No effective strategies for minimizing nephrotoxicity related to GEN therapy are available [7]. Naturally occurring agents have recently emerged as potential alternative resources for discovering new drugs [9]. The utilization of natural plant extract PTL to alleviate GEN-induced nephrotoxicity and the possible underlying mechanisms were studied. Our study revealed that PTL-phytosomes represent a promising therapeutic agent against kidney injury caused by GEN therapy. This was attributed to PTL-phytosomes’s antioxidant, anti-inflammatory, and anti-apoptotic properties. To our knowledge, this is the first research to demonstrate the renoprotective impact of PTL-phytosomes against GEN-induced nephrotoxicity in vivo.

PTL-phytosomes were prepared as a nanocarrier delivery system to improve their bioavailability. The particle size was 407.4 nm, with an excellent morphological structure. Consistent with our results, many natural products in phytosome formulation have biological activities in different diseases [44]. It was demonstrated that curcumin phytosomes had important antioxidant properties in kidney dysfunction patients [45]. Indeed, the current study revealed that administered PTL-phytosomes improved kidney function markers more significantly than administered PTL on GEN-treated rats. Utilizing photo-phospholipid complexes (phytosomes) is a strategy to enhance the oral bioavailability of active constituents. This is attributed to hydrogen-bond interactions between phospholipids and active members that contribute to generating phyto-phospholipid complexes as an integral part [40].

Moreover, this chemical bond improved drug encapsulation and stability profile [46]. Phytosomes increased phytoconstituent absorption and bioavailability. This leads to improved therapeutic effects [47].

Our results showed that GEN treatment significantly produced nephrotoxicity in rats, as evidenced by an increased kidney-to-body weight ratio. This was associated with an elevation in urea, creatinine, and cystatin C serum levels, indicating worsening kidney function. Consistently, exposure to GEN produced noticeable glomerular and tubular histopathological changes in rats’ kidneys. These results agreed with previous studies [8,48,49]. We found that oral administration of PTL-phytosomes (5 and 10 mg/kg/day) for seven days significantly improved kidney function by recovering all mentioned markers to normal levels and preserving the kidney histological picture. Similarly, a previous study reported that intraperitoneal (IP) administration of PTL (3 mg/kg/day) for four days lowered serum creatinine and improved kidney structure abnormalities in a cisplatin-induced renal damage rat model [21]. Another study revealed that IP administration of PTL (1 mg/kg) every other day for eight weeks alleviated glomerular hypertrophy and mesangial matrix enlargement in type 2 diabetic nephropathy in mice [50].

It is well established that GEN accumulation in the epithelial cells of the proximal tubules leads to structural and functional degradation of the cell membranes, mitochondria, and lysosomes, resulting in GEN-induced nephrotoxicity [51]. KIM-1 is a reliable marker for proximal tubular injury in experimental animals and human kidney disease. KIM-1 is a transmembrane tubular protein undetectable in healthy kidneys but is highly expressed in the proximal tubular cells after nephrotoxic or ischemia injury [52]. We found that GEN treatment upregulated KIM-1 expression in the rats’ kidneys, consistent with previous reports [12,53]. Interestingly, PTL-phytosomes treatment at both doses attenuated KIM-1 expression in the rats’ kidneys. Consistently, a previous study revealed alleviating kidney damage with naturally occurring dietary substances through suppressed KIM-1 expression in rats [54].

Oxidative stress is an important pathophysiological mechanism associated with GEM-induced renal damage [55]. It is primarily mediated by excessive ROS production, which disrupts the antioxidant state’s homeostasis [13] and damages numerous biological targets, particularly nucleic acids, lipids, and proteins [56]. Indeed, the involvement of ROS in promoting lipid peroxidation in the kidney has been well-established [10]. It has also been responsible for histopathological abnormalities in the renal tubules and glomeruli [57,58]. The current study demonstrates that GEN treatment induced lipid peroxidation by increasing MDA levels and inhibiting antioxidant enzyme activities, including SOD and CAT, in rats. These results are consistent with previous studies [59,60]. PTL-phytosomes treatment at both doses alleviated GEN-oxidative stress by restoring MDA, SOD, and CAT to normal levels. These results aligned with earlier research reporting that PTL possesses antioxidant activities in various cell types [61,62,63]. It has also been documented that IP administration of PTL (3 mg/kg) for four days suppressed cisplatin-induced oxidative stress by decreasing MDA levels in rats [21]. Moreover, PTL (0.5–1 mg/kg/daily) treatment for three days ameliorated intracerebral hemorrhage-induced oxidative stress in rats by decreasing the levels of ROS and increasing antioxidant activities [20].

It is well known that upregulation of the Sirt1 expression level activates the downstream Nrf2-Keap1 anti-oxidative signaling pathway. Research has proven that Sirt-1 responds sensitively to oxidative stress. It protects against renal damage due to its high expression level in the kidney tissue [64]. In agreement with previous studies, we observed that GEN treatment reduced Sirt-1, Nrf2, NQO1, and HO-1 protein expressions, as measured using the Western plot analysis [65,66,67]. The anti-oxidant effects of PTL were proposed to be exerted by activating Nrf2 and its target genes, NQO1 and HO-1 [18,63]. In the current study, we found that PTL-phytosomes protected against the generated oxidative stress in kidney tissues by raising Sirt-1, Nrf2, NQO1, and HO-1 protein expressions. Therefore, PTL treatment markedly attenuated the deterioration caused by GEN on these pathways. However, to elucidate whether the anti-oxidative effects of PTL are exerted via the Sirt1 and Nrf2-Keap1 signaling pathways, further studies must be conducted in an Nrf2-deficient condition.

The transcription factor NF-κB is one of the signaling pathways involved in GEN-induced nephrotoxicity and plays a crucial role in activating inflammatory responses [68]. GEN-mediated oxidative stress activates the NF-κB pathway, releasing NF-κB from the NF-κB/IκB complex and translocating it into the nucleus to activate the transcription of pro-inflammatory genes [69]. The current study revealed that the expression of NF-κB (p65) and pro-inflammatory cytokines, including IL-6, COX-2, and TNF-α, are augmented in GEN-treated rats. This is in line with previous studies [59,70]. TNF-α and IL-6 are the most versatile cytokines influencing inflammation and uncontrolled cell proliferation. In this sense, suppressing TNF-α and IL-6 might be an effective strategy against nephrotoxicity [27]. As expected, PTL-phytosomes treatment attenuated the inflammatory response by downregulating NF-κB (p65), IL-6, COX-2, and TNF-α expression. This is in agreement with the anti-inflammatory effects of PTL, which was found to be mediated through the inhibition of NF-κB expression in a cisplatin-induced renal damage rat model [21] and in animal models of different diseases, including type 2 diabetes [71], atherosclerosis [72], colitis [73,74], pulmonary fibrosis [75], and myocardial injury [76]. Moreover, a previous report established that PTL could significantly reduce IL-6, TNF-α, and COX-2 in rats’ brains [77]. It has also been demonstrated that PTL alleviated autoimmune neuritis by suppressing TNF-α expression in rats [78]. Additionally, the anti-inflammatory activity of PTL has been verified in multiple in vitro studies [28,79]. These anti-inflammatory activities of PTL are correlated with its chemical structure, which has α-methylene γ-lactone moiety. This component potentially interacts with particular NF-κB pathway sites [80].

Apoptosis induction is another important cytotoxic mechanism contributing to GEN-induced nephrotoxicity [81]. This mechanism is mediated by GEN-induced ROS, mitochondrial dysfunction, and NF-κB activation [82]. In this pathway, the balance between Bax and Bcl-2 proteins appears vital in controlling cell apoptosis and viability [83]. Upon stimulation of apoptosis, Bax acts as a pro-apoptotic protein, while Bcl-2 acts as an anti-apoptotic protein [84]. The present study revealed that GEN induced apoptosis by upregulating Bax and downregulating Bcl-2 mRNA expression, thereby elevating the Bax/Bcl-2 ratio in the rat’s kidney. These findings are supported by previous reports [84,85]. However, this imbalance of apoptosis proteins was significantly alleviated by PTL-phytosomes treatment that suppressed Bax and enhanced Bcl-2 mRNA expression, lowering the Bax/Bcl-2 ratio in the rat’s kidney. Previous research demonstrated that PTL decreased Bax expression and increased Bcl-2 expression in vitro [63]. Several studies have also established that the PTL has an antiapoptotic effect in other cells, including rat glomerular visceral epithelial cell line [31,32].

## 4. Materials and Methods

### 4.1. Chemicals and Drugs

PTL was bought from Salaus Nutra INC (Xi’an, China) with a purity of 99.83%. GEN was gifted from SPIMACO (Al-Qaseem, Saudi Arabia). All other chemicals were purchased commercially at high analytical quality.

### 4.2. Animals

All methods were authorized by the Research Ethics Committee at King Abdulaziz University’s Faculty of Pharmacy in Jeddah, Saudi Arabia (Reference No. PH-1443-41). Thirty-six adult male Wistar rats (175–230 g, 10–12 weeks old) were used in the study. Animals were kept in the animal care facility at the Faculty of Pharmacy at King Abdelaziz University. Animals were maintained at controlled room temperature, relative humidity (50–55%), and a twelve-hour light/dark cycle. Standard pellets and water were fed to the animals.

### 4.3. Preparation of PTL-Phytosomes

Phytosomes is a lipid-based nanoparticle technology that improves the pharmacokinetic properties of bioactive extracted from plants. Intercalation between the phytoconstituent and the polar component of the lipid is the primary mechanism for forming the phytosomes. PTL-phytosomes were prepared by the solvent evaporation method [86]. Briefly, PTL (99.2 mg) and phosphatidylcholine (376.8 mg) (soybean lecithin origin, content 90%) were dissolved in ethanol (30 mL) and chloroform (30 mL) by stirring until completely dissolved. The organic solvent (ethanol) was then removed by evaporation at 40 °C for 30 min with rotavapor. Then, a thin layer of phospholipid mixture was formed and hydrated with 80 mL of distilled water. Then, the mixture was sonicated with an ultra-wave sonicator for 30–40 min. Finally, the sample was taken to determine particle size and further analyzed.

### 4.4. Characterization of PTL-Phytosomes

#### 4.4.1. Particle Size

Dynamic light scattering was used to measure the particle size of PTL-phytosomes (Zetasizer, Nano-ZS, Malvern Company, Worcestershire, UK). The sample dispersion was diluted with Milli-Q water before the examination. The value was expressed as z-average size ± SD.

#### 4.4.2. Transmission Electron Microscopy

Morphological features of PTL-phytosomes were analyzed using Titan 80-300 TEM (Thermo Fisher Scientific, Waltham, MA, USA) at Electron Microscopy Lab, King Abdullah University of Science and Technology, Thuwal, Saudi Arabia. A drop of prepared PTL-phytosomes was placed on a carbon-glazed grid, allowed to form a thin film, and TEM images of PTL-phytosomes were taken using a OneView camera (Gatan Company, Pleasanton, CA, USA).

### 4.5. Experimental Design

The GEN-induced nephrotoxicity rat model was developed by a daily IP injection of 100 mg/kg GEN for seven consecutive days, according to previous experimental models [14,27]. A comparative study was conducted to evaluate the protective effects of PTL and PTL nano-formulations (PTL-phytosome) against GEN-induced nephrotoxicity in rats, as shown in Figure S1. As expected, PTL-phytosome-cotreated rats showed a significant renoprotective impact compared to the PTL-cotreated group. This could be attributed to the enhanced oral bioavailability. The dosage of PTL-phytosomes was determined based on a preliminary experiment in which several dosages of PTL-phytosomes ranging from 5, 10, and 15 mg/kg were tested. Both of the higher doses exhibited the same degree of nephroprotective activity. It was demonstrated that PTL 10 mg/kg is protective in other animal models [72,73].

The animals were divided into six groups (*n* = 6) in a random manner. Group 1 (Control): no drugs or solvents were administered to the rats. Group 2 (Vehicle): rats received oral nano-formulations plain vehicle. Group 3 (PTL-phytosomes alone): rats received oral PTL-phytosomes (10 mg/kg). Group 4 (GEN alone): rats received an IP dose of GEN (100 mg/kg). Group 5 (PTL-phytosomes + GEN): rats received a low oral dose of PTL-phytosomes (5 mg/kg), then were injected with IP GEN (100 mg/kg). Group 6 (PTL-phytosomes + GEN): rats received a high oral dose of PTL-phytosomes (10 mg/kg), then were injected with IP GEN (100 mg/kg).

Twenty-four hours after the last dose, the rats were weighed and anesthetized by IP injection of 80 mg/kg ketamine with 80 mg/kg xylazine. Blood samples were collected from the retro-orbital plexus and centrifuged for 10 min at 3000 rpm. The serum samples were kept at −80 °C for further analysis. The kidneys were removed and rinsed with an ice-cold NaCl isotonic solution (0.9%). The kidneys were weighed, and the relative kidney-to-body weight ratio was calculated as described previously [60]. The rat’s left kidney was fixed in 10% neutral formalin buffer for histological and immunohistochemical analysis. The rat’s right kidney was quickly stored in liquid nitrogen and kept at −80 °C for biochemical and RT-PCR analysis.

### 4.6. Evaluation of Renal Function Markers

To monitor kidney function, commercial kits to measure serum levels of creatinine (Cat. No. E4370-100, BioVision, Milpitas, CA, USA), urea (Cat. No. ab83362, Abcam, Cambridge, UK), and cystatin C (Cat. No. CSB-E08385r, CUSABIO, Fannin, TX, USA) were used.

### 4.7. Histopathological Study

The rat’s left kidney tissue was embedded in paraffin after being fixed in 10% neutral formalin buffer. The kidney tissues were cut into slices (5 μm) and stained with H&E stain to distinguish significant histopathological changes. Then, the PAS stain was used to determine the kidney’s mesangial matrix and the glomerular basement membrane. The histopathological alterations were examined to investigate the degree of kidney injury, such as tubular necrosis, infiltration of inflammatory cells, tubular dilation, cast formation, thickened glomerulus basement membrane, and capillary tufts. Three researchers evaluated the kidney injury independently and scored blindly, as presented in Table 1, using a previously described protocol [59]. Next, MT and SR stains were used to differentiate collagen fibrils and increase their amount between tubules, indicating interstitial fibrosis. Collagen deposition was quantified using ImageJ, 1.8.0_345, NIH, Bethesda, MD, USA. All stains were used to assess and examine the morphological changes caused by GEN-induced nephrotoxicity and the effect of the PTL-phytosomes. The slides were analyzed using an electric light microscope (Carl Zeiss Axiostar plus, Oberkochen, Germany).

### 4.8. Evaluation of Oxidative Status Markers

The homogenate of kidney tissues was used to evaluate oxidative stress markers. Kits for determining MDA (Cat. No. 10009055), SOD (Cat. No. 703002), and CAT (Cat. No. 707002) were obtained from Cayman^®^ Chemical (Ann Arbor, MI, USA).

### 4.9. Western Blot Analysis

The kidney tissues were lysed in RIPA lysis buffer with protease and phosphatase inhibitors (Cat. No. P0001, Sigma-Aldrich, St. Louis, MO, USA). The protein concentration of the supernatant was measured with a BCA protein assay kit (Biovision Inc. Milpitas, CA, USA). The protein was separated using sodium dodecyl sulfate-polyacrylamide gel and transferred onto polyvinylidene difluoride membranes (Bio-Rad Laboratories, Hercules, CA, USA). The membranes were thereafter blocked with 5% milk in 0.1% TBS-Tween20 for 1 h at room temperature, washed 3 times, and incubated overnight at 4 °C with the following primary antibodies: Sirt-1 (sc-74465), Nrf2 (Cat. No. MBS9608128, San Diego, CA, USA), NQO1 (Cat. No. sc-376023), and HO-1 (Cat. No. sc-390991, Dallas, TX, USA). After the membranes were washed three times, the membranes were incubated at room temperature for 1 h with the secondary antibody conjugated to horseradish peroxidase (Cat. No. ab205718, Abcam, Cambridge, UK). The membranes were visualized using ChemiDoc MP Imaging System with Image Lab Software (Bio-Rad Laboratories, Dubai, United Arab Emirates), and the proteins were quantitated with ImageJ, 1.8.0_345, NIH, Bethesda, MD, USA.

### 4.10. Immunohistochemical Analysis

Kidney tissue sections were deparaffinized by immersing them in a xylene solution. Then the sections were rehydrated by gradually decreasing the ethanol concentration. Next, the sections were heated for 10 min at 95–100 °C in 10 mM sodium citrate buffer, pH 6.0, before being incubated for 2 h in 5% bovine serum albumin in tris-buffered saline. For the chromogenic staining stage, the slides were stained with a Mouse and Rabbit Specific HRP/DAB Detection Kit (Abcam, Cambridge, UK). Then, slides were incubated with primary antibodies against IL-6 (Cat. No. ab9324, Abcam, Cambridge, UK), TNF-α (Cat. No. ab205587, Abcam, Cambridge, UK), COX-2 (Cat. No. ab179800), NF-κB (p65) (Cat. No. ab194726, Abcam, Cambridge, UK) and KIM-1 (Cat. No. MBS175125, San Diego, CA, USA). Image analysis was performed on three sections of tissue from each rat using the Image J analysis software (NIH, Bethesda, MD, USA).

### 4.11. Real-Time Polymerase Chain Reaction (RT-PCR)

RNA isolation reagent (Trizol) was utilized to isolate total RNA from kidney tissues [87]. Then the RNA was converted into cDNA using the Revert-aid cDNA synthesis kit (Cat. No. 205113, Qiagen, Germantown, MD, USA). RT-PCR was performed using the following kit: Cat. No. 180830 (Qiagen, Germantown, MD, USA). The expression of Bax and Bcl-2 was measured compared to β-actin. GAPDH was used as a housekeeping control gene. After the PCR amplification, delta-delta Ct (^ΔΔCt^) was used to calculate the expression of each target gene by subtracting GAPDH Ct from each sample Ct [88]. The list of primer sequences is shown in Table S1.

### 4.12. Statistical Analysis

GraphPad Prism 9.4.1 (GraphPad Software, San Diego, CA, USA) was utilized to perform a one-way ANOVA. This is followed by Tukey’s test for identifying differences between groups, and data are presented as means ± SD; *p* < 0.05 was considered statistically significant.

## 5. Conclusions

The current study provides a comprehensive understanding of the therapeutic potential of PTL-phytosomes in GEN-induced nephrotoxicity in rats and the possible mechanisms involved. These protective effects were mediated by activating the Sirt1, Nrf2, OH-1, and NQO1 axis. These effects were dose-dependent and were more pronounced in a higher dose of PTL. These protective effects were mediated by activating the Sirt1, Nrf2, OH-1, and NQO1 axis. Considering these findings and the excellent tolerability and safety of PTL, it is applicable to use this plant extract as a protective agent in patients receiving GEN without compromising the efficacy of bactericidal therapy.

## Figures and Tables

**Table 1:** The scoring system for each kidney sample.

Scores	Severity of Lesions
0	No pathological change
+1	Mild change
+2	Mild to moderate change
+3	Moderate change
+4	Moderate to severe change
+5	Severe pathological change

## Data Availability

Data are contained within the article.

## Supplementary Materials

The following supporting information can be downloaded at: https://www.mdpi.com/article/10.3390/molecules28062741/s1, Table S1: List of primers used for RT-PCR, Figure S1: The protective effects of PTL and PTL-phytosome on the kidney function markers.

## Sample Availability

Samples of the compounds Parthenolide are available from the authors.

## Abbreviations

GEN, gentamicin; PTL, parthenolide; KIM-1, kidney injury molecule-1; MDA, malondialdehyde; SOD, superoxide dismutase; CAT, catalase; Sirt-1, sirtuin 1; Nrf2, nuclear factor erythroid-2-related factor-2; NQO1, NAD(P)H quinone dehydrogenase 1; HO-1, heme oxygenase-1; Keap1; Kelch-like ECH-associated protein 1; IL-6, cytokines interleukin-6; TNF-α, tumor necrosis factor-alpha; NF-κB, nuclear translocation of nuclear transcription factor-kappa B; PDI, polydispersity index; TEM, transmission electron microscopy; H&E, hematoxylin and eosin; PAS, periodic acid-schiff; MT, mason’s trichrome; SR, sirius red; OD, optical density; COX-2,cyclooxygenase-2; Bax Bcl-2-associated X-protein; Bcl-2, B-cell lymphoma 2; RT-PCR, real-time polymerase chain reaction; IP, intraperitoneal.
