# Supplementary material for: Parthenolide Phytosomes Attenuated Gentamicin-Induced Nephrotoxicity in Rats via Activation of Sirt-1, Nrf2, OH-1, and NQO1 Axis
Source: Molecules. 2023 Mar 17;28(6):2741. doi: 10.3390/molecules28062741 (PMC10053629; doi:10.3390/molecules28062741)
Supplement: Supplementary file 1 [file molecules-28-02741-s001.zip › molecules-2206351-supplementary.pdf]

Supplementary Materials

Table S1. List of primers used for RT-PCR.

	Forward Primer	Reverse Primer	Accession Number
Bax	5′CCTGAGCTGACCTTGGAGCA	5′GGTGGTTGCCCTTTTCTACT	U32098.1
Bcl2	5′TGATAACCGGGAGATCGTGA	5′AAAGCACATCCAATAAAAAGC	NM_016993.1
β-Actin	5′TCCGTCGCCGGTCCACACCC	5′TCACCAACTGGGACGATATG	NM_031144.3

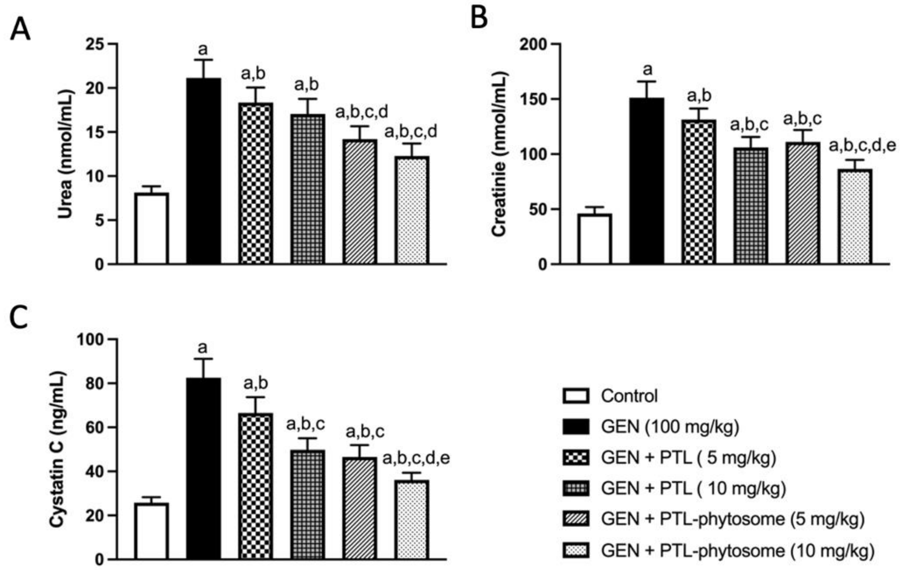

Figure S1. The protective effects of PTL and PTL-phytosome on the kidney function markers. Serum levels of kidney function markers, including (A) urea, (B) creatinine, and (C) cystatin C, were elevated in GEN-treated rats while decreased in both PTL and PTL-phytosomes co-treated rats. However, PTL- pyrosomes-cotreated rats (5 or 10 mg/kg) showed a significant reduction in the serum levels of kidney function markers compared to the PTL-cotreated group (5 or 10 mg/kg). Data are expressed as Mean ± SD (n = 6). a: significant when compared to control at $p < 0.05$; b: significant when compared to GEN-alone at $p < 0.05$; c: significant when compared to GEN + PTL (5 mg/kg) at $p < 0.05$; d: significant when compared to GEN + PTL (10 mg/kg) at $p < 0.05$; e: significant when compared to GEN + PTL-phytosomes (5 mg/kg) at $p < 0.05$.
